# Sexual selection of male parental care in giant water bugs

**DOI:** 10.1098/rsos.150720

**Published:** 2016-05-04

**Authors:** Shin-ya Ohba, Noboru Okuda, Shin-ichi Kudo

**Affiliations:** 1 Center for Ecological Research, Kyoto University, Otsu 520-2113, Japan; 2 Department of Biology, Naruto University of Education, Naruto, Tokushima 772-8502, Japan

**Keywords:** Belostomatidae, egg-bearing, mate choice, paternal care, sexual selection

## Abstract

Paternal care can be maintained under sexual selection, if it helps in attracting more mates. We tested the hypothesis in two giant water bug species, *Appasus major* and *Appasus japonicus*, that male parental care is sexually selected through female preference for caring males. Females were given an opportunity to choose between two males. In the first test of female mate choice, one male carried eggs on its back, while the other did not. The egg status was switched between these two males in the second test. The experiment revealed that females of both species preferred caring males (i.e. egg-bearing) to non-caring males. Nonetheless, the female mate preference for egg-bearing males was stronger in *A. major* than in *A. japonicus*. Our results suggest that sexual selection plays an important role in maintaining elaborate paternal care in giant water bugs, but the importance of egg-bearing by males in female mate choice varies among species.

## 1. Introduction

Evolution of post-zygotic paternal investment is an interesting but controversial topic in evolutionary biology [1,2]. There is a growing body of evidence that paternal care contributes to reproductive success by enhancing the survival and development of offspring [3–5]. Nonetheless, paternal care is less common than maternal care [3–5]. There have been repeated attempts to explain the reason for sex differences in parental care. For example, female promiscuity can cause high uncertainty of paternity, and thereby lead to decreased benefits of paternal care [1,6]. In addition, parental individuals usually have fewer re-mating opportunities due to a reduced ability to attract new mates while caring for offspring [7], and such a mating cost can be greater for males than for females, in which case males are not selected to invest in care [1,6].

Advances in behavioural ecology have proposed the idea that sexual selection can drive the evolution of male parental care [8]. While female mate preferences are unlikely to drive the emergence of paternal care, they may impact the maintenance and further evolution of care behaviours. Paternal care may enhance male reproductive success through increased mating opportunities, when females are attracted to the direct benefit of increased offspring survival [9]. Females that prefer caring males may also derive indirect genetic benefits, if the caring males have good genes to overcome the cost of parental care [8]. In such species, males may in turn advertise their parental intent to females [10]. The sexual selection hypothesis of evolution of paternal care has often been tested in fish [11–13], because paternal care is most commonly observed in fish species [4,5]. This hypothesis has rarely been tested in other taxonomic groups that exhibit paternal care. However, comparisons among phylogenetically independent taxa will deepen our understanding of the evolution of paternal care, enabling a more general application of the sexual selection hypothesis [14,15].

In arthropods, exclusive male care is rare; however, it has been observed in the case studies of 13 phylogenetically independent taxa [8,16]. These taxa provide good opportunities for researchers to examine the relative importance of natural and sexual selection in the maintenance of paternal care [8]. Only a few empirical studies have explicitly tested arthropod female preference for caring males under carefully controlled conditions [16,17]. The conclusions of these studies are inconsistent: female preference for caring males was observed in assassin bugs [18] and harvestmen [19], whereas sea spider females preferred non-caring males over males caring for eggs [20]. It would be premature to draw a general conclusion from these studies because proper laboratory experiments have not been designed to distinguish the effects of egg-bearing (‘caring’ in males) from those of other characteristics reflecting male quality [19].

In this study, we aimed to test the sexual selection hypothesis of maintenance of paternal care. To this end, we used giant water bugs (Heteroptera: Belostomatinae), which represent classic examples of paternal care systems in arthropods. Within this subfamily, all species exhibit exclusive paternal care for eggs, which females lay on the male's dorsum [21]. Paternal behaviour, i.e. brood pumping, brood stroking and surface brooding, is essential to egg development and survival [21], while this paternal care incurs a cost in terms of foraging efficiency [22], mobility [23] and longevity [24]. To examine female mate preference, we conducted experiments in which the caring status of males was carefully controlled, using two congeneric species: *Appasus major* and *Appasus japonicus*.

## 2. Material and methods

*Appasus major* (body length 20–26 mm) and *A. japonicus* (body length 16–21 mm) are widely distributed in Japan, excluding the Ryukyu Islands. *Appasus major* prefers cooler wetlands, and the local population density of this species is higher than that of *A. japonicus* [25,26]. *Appasus* species are iteroparous in both sexes. Egg brooding males occur from April to August, and newly adults emerge from July to August. Longevity of most adults is within a year, but some survive beyond two winters. Previous reports showed that *A. major* and *A. japonicus* were fundamentally univoltine (having one generation in a year) [27–29], but sometimes newly emerged adults reproduce before overwintering in the warmer habitat [26,30]. The populations of both species used in this study were univoltine (S. Ohba 2010, unpublished data).

Female *Appasus* lay their eggs on the back of the male, who broods them until hatching. An egg pad is laid on the back of the male by multiple females. Egg development periods depend on water temperature; they can last about a month in spring and about a week in mid-summer. After all eggs hatch, the male again mates with some females, and the females lay egg pads on his back within a few days [27]. Males care for more than one clutch (egg pad) under field conditions. Males care for a maximum of four clutches [27] (mean 1.9, *n* = 71, S. Ohba 2007, unpublished data) in *A. major*, and for a maximum of eight clutches (mean = 1.9, *n* = 148) in *A. japonicus*, within a breeding season [29]. In addition, each egg pad on the back of a male can be formed by multiple females in both *A. major* and *A. japonicus*.

### 2.1. Behavioural observation of female choice

*Appasus major* and *A. japonicus* were collected from irrigation channels in Hyogo, Japan. Nymphs were reared under natural environmental conditions (see the electronic supplementary material, S1) and newly emerged adults were used for the experiment (40 males and 43 females of *A. major*, and 52 males and 45 females of *A. japonicus*). Each adult was assigned a different identification number on the thorax for individual identification using a paint marker and was provided with crickets (*Gryllus bimaculatus*) ad libitum.

Prior to the mate choice experiment, the participating males were prepared as follows: 10 males were placed in a breeding aquarium (60 × 45 cm, water depth 5 cm) together with 10 females; the females were added only for egg deposition on the back of males for up to 24 h under laboratory conditions (25.0°C, 16 L : 8 D cycle). These spawning females were never used for the subsequent female mate choice experiments. When these males carried more than 10 eggs on their back, we picked up the males from the aquarium and manipulated the number of eggs by removing with forceps. The males were divided into two experimental groups: ‘caring males’, which carried 10 eggs (excess eggs were removed), and ‘non-caring males’, from which all eggs were removed. This manipulation ensured that both caring and non-caring males showed parental intent and were affected by the removal of eggs. Males fully loaded with eggs do not show courtship behaviour and reject female spawning, and thus, the presence of only 10 eggs on their back enabled the caring males to receive additional eggs from females. They were kept isolated in a plastic cup until use in the female mate choice experiments (described below) within 12 h of egg manipulation.

**Figure 1.**
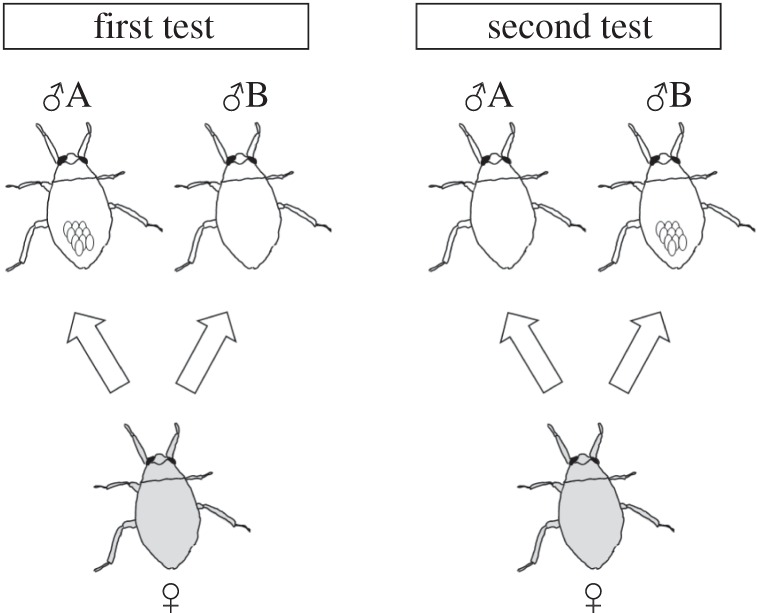
Experimental design for female mate choice.

In the first test of female mate choice, one caring male, one non-caring male and one female with mature eggs (judging by her greenish and swollen abdomen) were randomly selected and placed in a container (8.0 × 12.5 × 5.5 cm, water depth 3 cm) 0.5 h before sunset. One and a half hours after the placement, we counted the number of eggs added to the back of each of these two males as a proxy of female mate preference [13,18,31]. If no oviposition occurred within the 1.5 h, then the two males and the female were returned to the plastic cup, and the experimental procedure was repeated for the same trio on the next day. The second test of female mate choice was conducted with the same trios as in the first test (figure 1). After the first test, all eggs were removed from the two males. The caring and non-caring males in the first test were introduced again into the breeding aquarium, where both of them were allowed to receive a new set of eggs from spawning females. The egg removal manipulation was alternated between these two males, i.e. individuals that served as caring males in the first test were assigned to non-caring male duty in the second test and vice versa. The same trio as in the first test was placed again into the container for the second test of female mate choice. In cases when any individual in a trio died before the second test, the data from this trio were excluded from the analysis. We obtained data from 15 trios of *A. major* and 22 trios of *A. japonicus*. The intervals between the first and the second test were 11.3 ± 1.53 d (mean ± s.d.) in *A. major* and 12.7 ± 2.43 d in *A. japonicus*.

To examine the effects of egg-bearing by males on female mate preference, we used a generalized linear mixed model (GLMM, the glmmADMB package) with a negative binomial distribution, incorporating the number of eggs received from female as a response variable. A GLMM was used here because the response variables show overdispersion [32]. Each female was considered as a random effect. Species (*A. major* and *A. japonicus*), ‘egg-bearing’ (caring versus non-caring), test (first versus second) and their interactions were incorporated into the GLMM as an explanatory variable. If the interaction was not significant (*p* > 0.05), it was removed from the final model (see also the electronic supplementary material, S2). For each species, and each test within each species, the Exact Wilcoxon signed-rank test (wilcox.exact, exactRankTests package) was used to compare female preference for caring or non-caring males. The overall significance level was adjusted using a sequential Bonferroni method [33].

### 2.2. Behavioural observation of displays of male courtship

**Figure 2.**
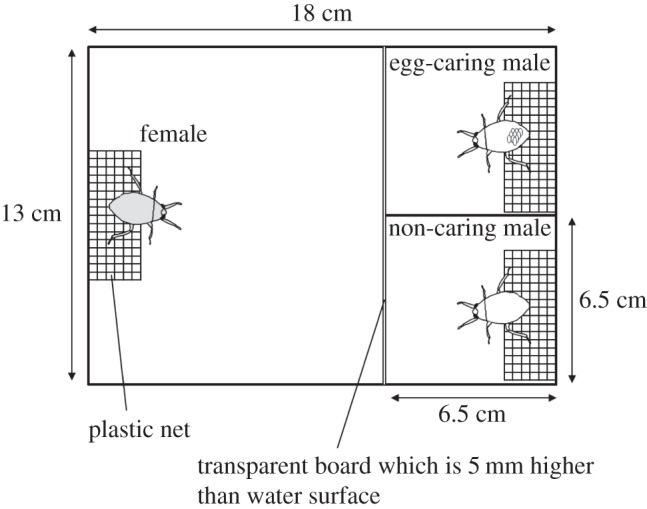
Behavioural observation of displays of male courtship.

In Belostomatidae, males assume an active role in courtship, performing rapid up-and-down movements called ‘pumping display’, which can be one criterion for female mate choice [34]. In this study, to test whether the possession of eggs affects male pumping display, we conducted certain behavioural observations (figure 2). According to the procedure of the laboratory experiment with female mate choice, a caring male, a non-caring male and a gravid female were randomly selected and placed into a plastic container (height 6 cm, water depth 3 cm, with a plastic 3 mm mesh for perching) 0.5 h before sunset for acclimation. We recorded the ‘pumping display’ by each of the two males for 30 min under twilight conditions (0.7 lx). In the container, a transparent partition enabled both males to communicate visually but not physically with the gravid female. We obtained data from 21 trios of *A. major* and 22 trios of *A. japonicus*. Data regarding the proportion of males performing the ‘pumping display’ (courtship rate) were analysed using a GLMM model (the glmmML package) with a binomial distribution for each species. The courtship rate was used as the response variable; ‘egg-bearing’ (caring versus non-caring) was used as explanatory variable; and each female was fitted as a random effect.

The statistical software R (v. 3.0.2) was used for all analyses [35].

## 3. Results

### 3.1. Behavioural observation of female choice

**Figure 3.**
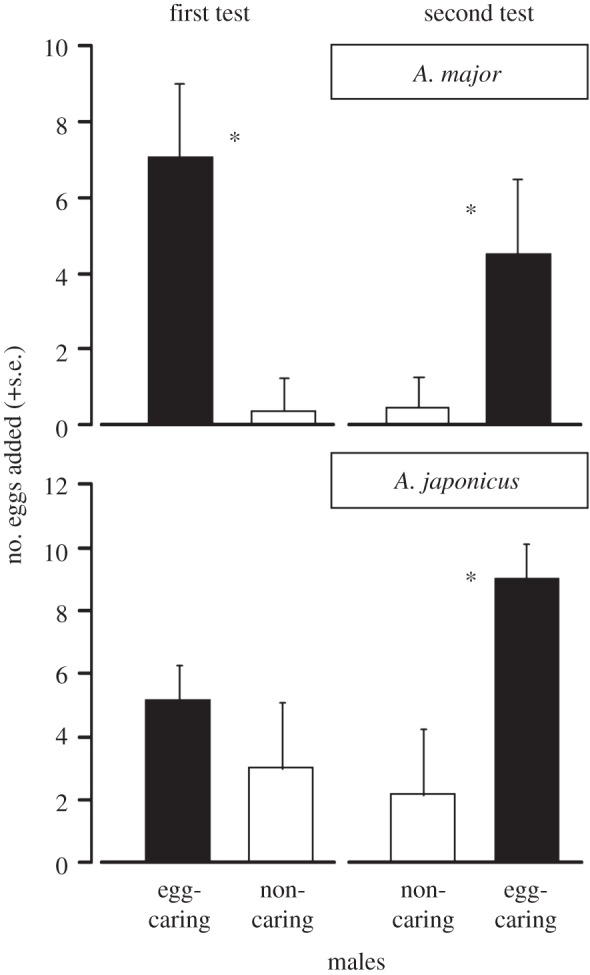
Comparison of the number of eggs added to the back of males. The number of eggs added to the back of egg-bearing males was significantly greater than that of non-caring males (*p < 0.05, Exact Wilcoxon signed-rank test with sequential Bonferroni method).

The interactions of egg-bearing behaviours in terms of species and test (species × egg-bearing × test, species × test and egg-bearing × test) were non-significant (see the electronic supplementary material, S3) and thus removed from the GLMM. The final model revealed that egg-bearing and species-by-egg-bearing interaction had a significant effect on the female mate preference (table 1). There is an interspecific difference in the degree of female mate preference for caring males between *A. major* and *A. japonicus*, with less choosiness in the latter (figure 3, see also the electronic supplementary material, S4). In a separate comparison for each test within each species, the female mate preference for egg-caring males was significantly greater than that for non-caring males. This was true for both tests on *A. major* (Exact Wilcoxon signed-rank test, first test, *V* = 53, *p* = 0.0059; second test, *V* = 98, *p* = 0.0022), and for the second test on *A. japonicus* (*V* = 152, *p* = 0.0198), but there was no significance in first test on *A. japonicus* (*V* = 111, *p* = 0.2779; figure 3).

**Table 1.** The results from the GLMM regarding the number of eggs added to the dorsum of each male under the laboratory conditions. Factors shown in italics are significant (*p* < 0.05).

Source	estimate	s.e.	*Z*	*p*-value
intercept	1.944	0.328	5.92	<0.001
species (S)^a^	−0.201	0.421	−0.48	0.633
egg-bearing (E)^b^	−*1*.*013*	*0*.*385*	−*2*.*63*	*0*.*009*
test (T)^c^	0.008	0.312	0.02	0.980
S by E	−*1*.*737*	*0*.*668*	−*2*.*60*	*0*.*009*

a The coefficient indicates the relative effect of egg-bearing *A. major* during test 1 compared with egg-bearing *A. japonicus* during test 1.

b The coefficient indicates the relative effect of non-caring male *A. japonicus* during test 1 compared with caring male *A. japonicus* during test 1.

c The coefficient indicates the relative effect of second test for egg-bearing *A. japonicus* compared with first test for egg-bearing *A. japonicus*.

### 3.2. Behavioural observation of displays of male courtship

The intensity of male pumping displays did not significantly differ between the caring and the non-caring males in both *A. major* (caring versus non-caring = 76.9% versus 84.6%, *z* = 0.302, *p* = 0.763, GLMM) and *A. japonicus* (caring versus non-caring = 50.0% versus 63.6%, *z* = 1.22, *p* = 0.222), suggesting that male courtship behaviour did not change as a result of the presence of eggs on the back.

## 4. Discussion

This study using *A. japonicus* and *A. major* shows that females choose males for mating and that the caring status of males is under sexual selection. The mate choice experiments reveal that females prefer caring males (i.e. egg-bearing males) to non-caring males (i.e. males free of eggs) in both species (figure 3), as reported previously for assassin bugs [18] and harvestmen [19]. Because the presence of eggs on the back does not enhance male pumping displays, it is unlikely that egg-related paternal behaviour attracts more females because of courtship behaviour.

Several possible perceived benefits have been suggested for females that prefer to mate with caring males. For example, males may increase their paternal effort when their clutch size increases through mating with multiple females [36] and females that add eggs can lower the risk of egg predation through the dilution effect [37]. *Appasus* females can also enjoy a direct benefit from choosing egg-bearing males. In Belostomatinae, males sometimes remove the egg pad from their back [38]. Such abandonment occurs more frequently in males with a smaller egg pad, which may bring a low return (benefit) relative to the cost of parental care [38]. Females, therefore, could prevent the abandonment of their own eggs by selecting males carrying eggs instead of males without eggs on their back. In fact, brooding can impose high costs on the male. For example, active egg-caring behaviour, such as brood pumping, appears to incur energetic costs. Egg-bearing reduces swimming and foraging abilities [22,23]. Males brooding eggs on their backs, which are conspicuous, often remain at the air–water interface; thus, they may suffer high predation risks [21].

It should be noted that the availability of egg-bearing males changes within the breeding season: at the beginning of a breeding season, there are no egg-bearing males. Various sexual selection regimes have been reported under such conditions among giant water bug species. In *Abedus indentatus*, females choose males that demonstrate longer pumping displays, which may indicate male parental ability [34]. By contrast, in *Belostoma lutarium*, males are likely to be the choosier sex, and it is the body weight of females that is under sexual selection [39]. Clarifying sexual selection and its target traits in the two species in the absence of egg-bearing males will be the subject of an upcoming project.

One of the key conditions for the evolution of male parental care is the ability of males to simultaneously care for eggs deposited by multiple females, i.e. they brood overlapping clutches (e.g. [15]); such polyandrous conditions will allow caring males to be under sexual selection. In *Appasus* species, the number of eggs brooded by a single male often exceeds the number of eggs that a single female can produce. In *A. major*, the maximum number of eggs produced by a female is 46 in the field, whereas that carried by a single male is 117 [27]. *Appasus japonicus* males receive up to 155 eggs in the field, whereas females never lay more than 50 eggs [40]. These data strongly suggest that *Appasus* males receive eggs from multiple females and thus brood overlapping clutches. This seems to be the case for other giant water bug species as well [41]. If male parental care is favoured by sexual selection, caring for offspring might benefit males without high paternity [2,8]. This may also be the case for *A. major*; in this species, on average, 28.4% of eggs on the back of a male were fertilized by other males in the field [42].

This study also reveals an interspecific difference between *A. major* and *A. japonicus* in the degree of female mate preference for caring males, with *A. japonicus* demonstrating less choosiness (figure 3). Females of *A. japonicus* showed a preference that increased over time, whereas those of *A. major* showed a constant preference. According to sexual selection theory, mate choosiness can be influenced by a variety of factors, such as variation in mate quality, mate encounter rate, sex ratios and predation risk [43]. In the wild, local population density is approximately twofold higher for *A. major* than for *A. japonicus* [25]. The high population density can increase the frequency of intersexual encounters, making females more selective. The sex ratio (the ratio of the number of adult males to the number of all adults) is 0.45 and 0.62 in *A. major* and *A. japonicus*, respectively, in July (mid-breeding season for this study population, S. Ohba 2007, unpublished data). The variation in mate availability is a factor that may explain the difference in female choosiness between these two species.

In conclusion, our results, together with the existing evidence, indicate that sexual selection plays an important role in the maintenance of elaborate paternal care in giant water bugs, but the importance of egg-bearing by males in female mate choice varies among species.

## Supplementary Material

ESM 1. Insect rearing method ESM 2. Selected models explaining the number of eggs added to the dorsum of each male. ESM 3. The GLMM (full model) regarding the number of eggs added to the dorsum of each male under the laboratory conditions. ESM 4. Female mate preference for egg-caring males

## Data accessibility

The data are available as the electronic supplementary material. Data available from the Dryad Digital Repository: http://dx.doi.org/10.5061/dryad.p4sk4.
