# Supplementary material for: Sexual selection of male parental care in giant water bugs
Source: R Soc Open Sci. 2016 May 4;3(5):150720. doi: 10.1098/rsos.150720 (PMC4892440; doi:10.1098/rsos.150720)
Supplement: ESM 1. Insect rearing method ESM 2. Selected models explaining the number of eggs added to the dorsum of each male. ESM 3. The GLMM (full model) regarding the number of eggs added to the dorsum of each male under the laboratory conditions. ESM 4. Female mate preference for egg-caring males [file rsos150720supp1.docx]

**Electronic supplementary material 1**

**Insect rearing method**

For *Appasus major*, the first instar nymphs were collected from irrigation channels in the western part of Hyogo, central Japan, in June 2010; adults that emerged and overwintered in 2010 were also collected from April to July 2011. For *A. japonicus*, 15 caring males were collected from the same study site as that of *A. major* in June 2010 to obtain hatched nymphs as experimental subjects.

For both species, the nymphs were reared individually in a plastic cup (diameter 100 mm, height 45 mm, water depth 2 cm, supplied with a piece of a plastic 5-mm mesh as a perching substratum) under natural environmental conditions (i.e. ambient air temperature and usual day length) until they reached the adult stage. The nymphs were provided with a variety of prey *ad libitum*: aquatic isopoda (*Asellus* spp.), aquatic snails (Lymnaeidae and Physidae), mosquito larvae (*Aedes* spp.), and artificial frozen chironomid larvae (Kyorin Co., Ltd., Tokyo, Japan).

From December 2010 to March 2011, emerging adults of *A. major* and *A. japonicus* were kept at 5–8°C for overwintering. Together with adults that emerged in nature and were collected, they were used for the experiment with female mate choice between April and July 2011 (40 males and 43 females of *A. major* and 52 males and 45 females of *A. japonicus*). Each adult was assigned a different identification number with a paint marker on the thorax for individual identification and was provided with crickets (*Gryllus bimaculatus*) *ad libitum*.

**Electric supplement material 2.**

Selected models explaining the number of eggs added to the dorsum of each male.

| Explanatory variable | AIC |
| --- | --- |
| **species (S) + egg-bearing (E) + test (T) + S*E** | **641.9** |
| species (S) + egg-bearing (E) + test (T) + S*E + S*T | 643.6 |
| species (S) + egg-bearing (E) + test (T) + S*E + E*T | 643.6 |
| species (S) + egg-bearing (E) + test (T) + S*E + S*T + E*T | 645.2 |
| species (S) + egg-bearing (E) + test (T) + S*E + S*T + E*T + S*E*T | 645.8 |
| Null model | 662.9 |

**Electric supplement material 3.**

The GLMM (full model) regarding the number of eggs added to the dorsum of each male under the laboratory conditions.

| Source | Estimate | S.E. | *z* | *P* |
| --- | --- | --- | --- | --- |
| Intercept | 1.628 | 0.371 | 4.39 | <0.001 |
| Species (S) ^†^ | 0.318 | 0.579 | 0.55 | 0.583 |
| Egg-bearing (E)^††^ | -0.544 | 0.530 | -1.03 | 0.305 |
| Test (T) ^†††^ | 0.565 | 0.521 | 1.08 | 0.278 |
| **S by E** | **-2.500** | **0.932** | **-2.68** | **0.007** |
| S by T | -1.014 | 0.820 | -1.24 | 0.216 |
| E by T | -0.889 | 0.752 | -1.18 | 0.237 |
| S by E by T | 1.520 | 1.308 | 1.16 | 0.245 |

^†^The coefficient indicates the relative effect of egg-bearing *A. major* during test 1 compared with egg-bearing *A. japonicus* during test 1

^††^The coefficient indicates the relative effect of non-caring male *A. japonicus* during test 1 compared with caring male *A. japonicus* during test 1.

^†††^The coefficient indicates the relative effect of 2nd test for egg-bearing *A. japonicus* compared with 1st test for egg-bearing *A. japonicus*.

**Electric supplement material 4.**

Female mate preference for egg-caring males was significantly greater than that for non-caring males in both species (Exact Wilcoxon signed rank test, *A. major*, *V* = 283.5, *P* < 0.0001: *A. japonicus*, *V* = 513, *P* = 0.0137), with less selectivity in the latter. The data of both 1st and 2nd tests were pooled for each species.
